# Relations between bedtime parenting behaviors and temperament across 14 cultures

**DOI:** 10.3389/fpsyg.2022.1004082

**Published:** 2022-11-24

**Authors:** Christie Pham, Eric Desmarais, Victoria Jones, Brian F. French, Zhengyan Wang, Samuel Putnam, Sara Casalin, Maria Beatriz Martins Linhares, Felipe Lecannelier, Soile Tuovinen, Kati Heinonen, Katri Raikkonen, Rosario Montirosso, Lorenzo Giusti, Seong-Yeon Park, Sae-Young Han, Eun Gyoung Lee, Blanca Huitron, Carolina de Weerth, Roseriet Beijers, Mirjana Majdandžić, Carmen Gonzalez-Salinas, Ibrahim Acar, Helena Slobodskaya, Elena Kozlova, Emine Ahmetoglu, Oana Benga, Maria A. Gartstein

**Affiliations:** ^1^Department of Psychology, Washington State University, Pullman, WA, United States; ^2^College of Education, Washington State University, Pullman, WA, United States; ^3^College of Psychology, Capital Normal University, Beijing, China; ^4^Bowdoin College, Brunswick, GA, United States; ^5^Department of Psychology, University of Leuven, Leuven, Belgium; ^6^Department of Neurosciences and Behavior, University São Paulo, São Paulo, Brazil; ^7^Faculty of Medical Sciences, Universidad de Santiago, Santiago, Chile; ^8^Department of Psychology and Logopedics, University of Helsinki, Helsinki, Finland; ^9^Department of Social Sciences, University of Tampere, Tampere, Finland; ^10^0-3 Centre for the at-Risk Infant, Scientific Institute, IRCCS Eugenio Medea, Lecco, Italy; ^11^Department of Child Development, Ewha Woman’s University, Seoul, South Korea; ^12^Ewha Social Science Research Institute, Seoul, South Korea; ^13^Department of Psychology, National Autonomous University of Mexico, México City, Mexico; ^14^Department of Cognitive Neuroscience, Radboud University Medical Center, Nijmegen, Netherlands; ^15^Department of Developmental Psychology, Radboud University, Nijmegen, Netherlands; ^16^Department of Child Development and Education, University of Amsterdam, Amsterdam, Netherlands; ^17^Department of Developmental Psychology and Education, University of Murcia, Murcia, Spain; ^18^Department of Psychology, Özyeğin University, Istanbul, Turkey; ^19^Research Institute of Neuroscience and Medicine, Novosibirsk State University, Novosibirsk, Russia; ^20^Department of Early Childhood Education, Trakya University in Edirne, Edirne, Turkey; ^21^Department of Psychology, Babes Bolyai University, Cluj-Napoca, Romania

**Keywords:** sleep, parenting behaviors, temperament, cross-cultural comparisons, toddlerhood

## Abstract

**Objectives:**

The present study examined parental sleep-supporting practices during toddlerhood in relation to temperament across 14 cultures. We hypothesized that passive sleep-supporting techniques (e.g., talking, cuddling), but not active techniques (e.g., walking, doing an activity together), would be associated with less challenging temperament profiles: higher Surgency (SUR) and Effortful Control (EC) and lower Negative Emotionality (NE), with fine-grained dimensions exhibiting relationships consistent with their overarching factors (e.g., parallel passive sleep-supporting approach effects for dimensions of NE).

**Methods:**

Caregivers (*N* = 841) across 14 cultures (M = 61 families per site) reported toddler (between 17 and 40 months of age; 52% male) temperament and sleep-supporting activities. Utilizing linear multilevel regression models and group-mean centering procedures, we assessed the role of between- and within-cultural variance in sleep-supporting practices in relation to temperament.

**Results:**

Both within-and between-culture differences in passive sleep-supporting techniques were associated with temperament attributes, (e.g., lower NE at the between-culture level; higher within-culture EC). For active techniques only within-culture effects were significant (e.g., demonstrating a positive association with NE). Adding sleep-supporting behaviors to the regression models accounted for significantly more between-culture temperament variance than child age and gender alone.

**Conclusion:**

Hypotheses were largely supported. Findings suggest parental sleep practices could be potential targets for interventions to mitigate risk posed by challenging temperament profiles (e.g., reducing active techniques that are associated with greater distress proneness and NE).

## Introduction

The importance of cultural context in child development has been long recognized, with related topics the subject of theoretical and empirical efforts. The “Developmental Niche” conceptual model, proposed by Super and Harkness (1986) has been particularly influential in framing links between culturally influenced parenting, sleep, and temperament development. This developmental socioecological framework construes the ecological context in which a child develops as three integrated subsystems: (1) the physical and social settings where the child resides/spends time; (2) cultural norms of parenting; and (3) parental psychology and practices (e.g., caregiver values/priorities and parenting behaviors; Super and Harkness, 1986; Harkness and Super, 1994). Through reciprocal effects, these subsystems work in tandem to shape the sociocultural interface between the child’s development and their environment, with components examined across a variety of cultures from Malaysia to Kenya to Bangladesh to the United States (for review see Harkness and Super, 1994). According to this conceptual framework, it is critical to examine factors that support healthy development, such as the role caregivers play in shaping sleep during early childhood, discerning their culturally based underpinnings.

The significance of sleep across childhood has been extensively documented, including effects on brain maturation (Scher, 2005; Sadeh, 2007; Touchette et al., 2007; Sadeh et al., 2014; El-Sheikh et al., 2017). Compromised sleep appears to be detrimental for neurobehavioral functioning, emotional reactivity and regulation, as well as risk for future psychopathology (Sadeh et al., 2014). Sleep patterns and temperament have been consistently linked (Sadeh and Anders, 1993; Jian and Teti, 2016). For instance, Negative Affectivity, an aspect of temperament, and its dimensions have been associated with sleep development and problems such as night waking from 6 to 12 months of age (Morales-Munoz et al., 2020). Child temperament, defined in terms of individual differences in self-regulation and reactivity (Rothbart and Derryberry, 1981; Rothbart et al., 1994), is important to study in its own right and because of implications for trajectories marked either by behavioral–emotional health and wellbeing or risk for symptoms/disorders. For example, temperament components of impulsivity and anger were related to externalizing problems, whereas fear linked to internalizing difficulties in 36-month-olds (Karreman et al., 2010). Furthermore, the associations between “difficult” temperament (e.g., negative mood, low adaptability, high intensity) and clinically significant externalizing behavior problems has been demonstrated in 3- to 7-year-old and 8- to 12-year-old children (Maziade et al., 1990; for a review of further connections see Sanson et al., 2004), showing stability throughout development. It is important to expand the existing literature by examining parenting factors (especially sleep-supporting behaviors, which can be altered or adjusted) and their links to temperament development across cultures, as understanding parental contributions provide targets for potential preventative efforts.

Children receive an extensive amount of exposure to their caregiver during bedtime (Sadeh et al., 2010) that gradually decreases with age. Studies examining the interaction between infant sleep and parenting sleep-related practices have shown that infants whose parents were present when they fell asleep were more likely to experience night waking compared to those who slept independently (Adair et al., 1991), and that co-sleeping in response to night waking also increased difficulties (Karraker, 2008). On the other hand, regularity of bedtime routines across the first year of life decreases sleeping issues overall (Sadeh et al., 2010), with lasting protective effects particularly at a higher “dose” of routine (i.e., with increased frequency; Mindell et al., 2015). Patrick et al. (2016) reported that more consistent bedtime routines were associated with better sleep outcomes for children from three to 5 years of age. More frequent night waking was correlated with parental presence and active soothing techniques, such as breastfeeding back to sleep, that varied significantly between cultures (Mindell et al., 2010). Maternal reliance on active soothing techniques has also been correlated with maintenance of sleep issues for children (Morrell, 1999), and parental beliefs regarding supporting child sleep were shown to vary cross-culturally (Mindell et al., 2010). Furthermore, cross-cultural differences in parental bedtime behaviors/practices during infancy and childhood (Giannotti and Cortesi, 2009; Mindell et al., 2010; Sadeh et al., 2010) have been reported and likely contribute to cross-cultural variability in toddler temperament. For example, Dutch parents have been described as emphasizing sleep promotion and structuring daily activities in a manner that provides maximum support for regular sleep patterns (e.g., Super et al., 1996).

Though various aspects of sleep have been studied widely with regard to temperament in early childhood, including sleep problems (Atkinson et al., 1995; Molfese et al., 2015; Baukiene and Jusiene, 2016), sleep/wake regulation (Scher et al., 1998), sleep duration (Berger et al., 2018), bedtime resistance (Wilson et al., 2015), and sleeping arrangements (Hayes et al., 2002), relations between parental efforts to support sleep and temperament have been studied less often, with mixed results (Halpern et al., 1994; Kelmanson, 1999), and not considering cultural influences/differences. This gap in the field is especially notable given established cross-cultural differences in both temperament (e.g., Gartstein et al., 2003, 2006, 2010; Montirosso et al., 2011; Gaias et al., 2012; Cozzi et al., 2013; Krassner et al., 2017; Desmarais et al., 2019) and parental sleep-supporting practices (e.g., Jenni and O’Connor, 2005; Mindell et al., 2010, 2013; Gartstein and Putnam, 2018). South Korean toddlers, for example, scored significantly higher on the temperament dimension of Effortful Control compared to United States toddlers, yet lower on Surgency (Krassner et al., 2017). When examining differences in sleep practices between these cultural groups, 57% of the predominantly Caucasian group promoted independent sleep for their infants, whereas in the predominantly Asian group this percentage dropped to 4% (Mindell et al., 2010). Furthermore, findings from the Joint Effort Toddler Temperament Consortium (JETTC) indicate that varying sleep-supporting techniques across cultures differentially correlated with child temperament (Gartstein and Putnam, 2018). Specifically, active sleep-supporting behaviors (e.g., walking, car ride, special activity) were associated with higher ratings of Surgency, Effortful Control, and Negative Emotionality whereas passive sleep-supporting techniques (e.g., talking, cuddling) were linked with higher Surgency and Effortful Control, but lower Negative Emotionality. Given these differences, it is crucial to study the interplay between sleep practices and temperament through a cross-cultural lens as this knowledge may inform culturally sensitive interventions aimed to mitigate developmental risk.

To operationalize temperament, the psychobiological construct is defined by three overarching factors across childhood: (1) Surgency (SUR), reflecting positive affect such as smiling and laughter, approach tendencies, activity, and enthusiasm, (2) Negative Emotionality (NE), capturing overall distress proneness, including in situations eliciting fear, anger, sadness, and discomfort, and (3) Effortful Control (EC), involving attention-based regulatory skills and enjoyment of calm activities (Rothbart et al., 2001; Gartstein and Rothbart, 2003; Putnam et al., 2006). Each of these factors independently contributes to predicting behavioral, achievement, and interpersonal outcomes, such as behavior problems, social competence, and academic performance (Lengua, 2006; Rothbart and Bates, 2006; Gartstein et al., 2012, 2016), and fine-grained dimensions (i.e., subscales) that make up the overall dimension should be considered in their own right. For example, fine-grained dimensions have demonstrated developmental trajectories that differed from those of their overarching factors (Gartstein and Hancock, 2019), and uniquely contribute to temperament profile/types (Garstein et al., 2017). Perhaps most importantly, fine-grained dimensions were shown to have distinctive relations with behaviors, such as sleep, critical to children’s health and development (e.g., Gartstein et al., 2014; Jian and Teti, 2016; Morales-Munoz et al., 2020). Specifically, Gartstein et al. (2014) found vocal reactivity and sleep problems to be negatively correlated, and Jian and Teti (2016) reported that smiling/laughter and vocal reactivity moderated relations between mother’s bedtime emotional availability and infant sleep time variation: infants demonstrating higher levels of these fine-grained attributes experienced more sleep time than others if their mothers were emotionally available at bedtime. Morales-Munoz et al. (2020) found that higher fear, a fine-grained dimension of Negative Affectivity, was independently related to more night waking in 12-month-olds.

Our study examines parental sleep-supporting practices during the transitional period of a sleep routine consolidation for toddlers (Sadeh and Anders, 1993; Iglowstein et al., 2003; Staples et al., 2015) in relation to temperament across 14 cultures using the JETTC dataset. The “Developmental Niche” model indicates that culturally influenced parenting promotes certain developmental tendencies. Thus, the present study advances previous work by utilizing multilevel models (MLM) to elucidate the effects of both between-and within-cultural differences in parental sleep techniques (i.e., active and passive) in relation to toddler temperament. That is, we assessed the effects of both culture-level mean differences in the use of active and passive sleep-supporting techniques as well as the effects of individual variation in sleep practices within cultures. We hypothesized that passive sleep-supporting techniques, but not active techniques, would be associated with higher SUR and EC as well as lower NE. Fine-grained temperament dimensions, not previously examined, were expected to exhibit patterns of relationships consistent with their overarching factors (e.g., parallel passive sleep-supporting approach effects for dimensions of NE). Because previous research has indicated both between-and within-culture effects for other aspects of development (e.g., Deater-Deckard et al., 2018) we anticipate obtaining support for both herein. This study further expands on the work reported by Gartstein and Putnam (2018) by examining cross-cultural differences through a more optimal analytic lens, and considering these relations at the critical fine-grained dimension level.

## Materials and methods

### Participants

Data for this project was collected from 2015 to 2017. JETTC sites were selected to capture a wide range of geographic regions with meaningful variability in cultural orientation (e.g., individualism versus collectivism) and culturally driven parenting practices. These were also sites where investigators were using Rothbart temperament instruments, thus translation efforts had already been undertaken and relationships required for data collection established (for further details on the JETTC sites please see Chapter 2 Putnam et al. (2018)). Across the JETTC sites (i.e., Belgium, Brazil, Chile, China, Finland, Italy, Mexico, Netherlands, Romania, Russia, Spain, South Korea, Turkey, United States), mean enrollment was 61 families, ranging from a low of 49 families in Chile to a high of 119 families in the Netherlands (Table 1). Of the 865 families who completed the study, 841 of them responded to the Daily Activities Questionnaire (DAQ; Gartstein and Putnam, 2018), which was the sample size for final models. These were families of children between 17 and 40 months of age (*M* = 26.88 months, SD = 5.65 months), approximately equal in representation of child gender (52% male). For all but two of the JETTC cultures, data were collected at a single site, and for the two cultures (the Netherlands and US) where data were collected from two locations, there were no significant differences (*p* > 0.05) between sites on the variables used in this study. As is common with cross-cultural research (Keller et al., 2006), recruitment strategies varied across sites and depended on the cultural viability of methods. In general, approaches included social media, websites for new parents, flyers distributed at child-care centers and pediatric medical offices, as well as in person efforts by research assistants (e.g., at Saturday Market). Families in this study primarily reflected middle socioeconomic status (Revised Duncan Sociometric Index, RDSI; Stevens and Featherman, 1981) and were considered to be representative of their respective communities. However, it is important to keep in mind that these JETTC families may not necessarily be representative of their respective cultures as a whole. The study was approved by the institutional review boards/ethics committees overseeing the research at each of the sites involved.

**Table 1:** Sample demographics by culture.

Culture	Child gender		Child age (in months)			Family socio-economic status (RDSI)^1^			Marital status (in percent)^2^				Maternal education (in years)			Maternal age (in years)			# of children in the household		
	F	M	Range	*M*	SD	Range	*M*	SD	Ma	Lt	Di	Si	Range	*M*	SD	Range	*M*	SD	Range	*M*	SD
US	49	39	17–36	25.6	5.8	10–97	50.3	26.2	92	7	1	0	9–24	17.2	2.3	23–46	33.1	4.47	1–6	1.7	1
Belgium	21	27	17–41	25.7	5.3	10–97	63.8	21.1	56	38	12	4	10–32	18.0	2.9	27–38	32.26	2.67	1–5	1.9	1
Brazil	23	28	18–38	29.4	5.6	15–96	56.9	24.2	82	12	0	6	11–37	18.3	4.9	22–43	32.90	4.55	1–3	1.4	1
Chile	21	28	17–41	27.3	7.2	10–97	49.7	28.3	62	15	2	21	12–28	18.1	4.9	17–41	28.54	7.11	1–4	1.8	1
China	30	24	19–36	26.4	4.7	15–97	58.7	29.9	87	13	0	0	8–23	15.6	3.6	21–40	30.11	3.99	1–2	1.2	1
Finland	24	31	18–40	27.6	5.7	10–97	61.6	20.8	62	30	2	6	12–26	17.7	2.6	24–41	33.57	3.87	1–4	1.5	1
Italy	24	28	17–36	26.6	4.9	15–97	61.9	20.6	77	23	0	0	11–25	17.2	3.1	30–48	37.15	3.72	1–5	1.7	1
Mexico	25	29	18–36	26.4	5.6	10–97	38.3	29.8	69	24	6	1	9–25	16.8	3.8	17–43	32.35	5.89	1–5	1.6	1
Netherlands	55	64	16–40	26.6	5.8	10–87	56.6	22.3	53	40	2	5	5–25	17.7	3.7	20–41	31.99	4.27	1–3	1.6	1
Romania	30	28	17–38	21.2	6.4	15–97	72.4	19.4	98	2	0	0	12–29	18.1	6.4	23–41	32.91	3.93	1–3	1.4	1
Russia	26	25	17–36	27.0	5.6	15–93	62.8	19.0	77	21	2	0	10–22	14.9	2.1	21–43	29.37	5.20	1–8	1.6	1
Spain	27	35	18–35	26.1	5.1	10–97	58.2	27.3	74	18	1	7	8–21	15.6	4.2	29–43	35.88	3.55	1–4	1.8	1
S. Korea	26	27	17–35	28.0	4.8	15–96	51.6	24.5	100	0	0	0	7–18	15.3	2.2	29–44	34.58	3.45	1–3	1.9	1
Turkey	25	34	16–36	27.7	5.6	10–97	50.5	26.1	92	7	1	0	9–24	14.4	3.9	19–46	31.78	5.46	1–4	1.4	1

1 RDSI: Revised Duncan Sociometric Index—an occupation based measure of social prestige, based on maternal occupations (Stevens and Featherman, 1981).

2 Ma, married; Lt, living together; Di, divorced; and Si, single.

Table adapted from Gartstein et al. (2018), with permission from Routledge.

### Measures

Temperament was measured using the Early Childhood Behavior Questionnaire (ECBQ; Putnam et al., 2006), based on the psychobiological temperament framework (Rothbart et al., 1994). This measure includes 201 items, comprising 18 fine-grained scales, in turn forming three overarching factors. These items are rated on a 7-point rating scale with responses that range from “1-Never” to “7-Always.” In general, higher scores reflect a greater quantity of the particular attribute, as observed by the parent. The first factor, labeled Surgency (SUR), consists of five subscales: impulsivity, activity level, high-intensity pleasure, sociability, and positive anticipation. The second factor, Negative Emotionality (NE), consists of eight subscales: discomfort, fear, motor activation, sadness, perceptual sensitivity, shyness, soothability, and frustration. The third and final factor, labeled Effortful Control (EC) consists of five subscales: inhibitory control, attention shifting, low-intensity pleasure, cuddliness, and attention focusing. For each JETTC site, translation of the ECBQ was carried out by the respective principal investigators with an author of the original ECBQ providing feedback on back-translated items. The ECBQ was originally designed for children 18-to 36-months of age, yet mild expansion of age range is typical for childhood temperament instruments, given that items remain developmentally appropriate (Putnam et al., 2014). Therefore, a subset of children between 15- and 18-months (*n* = 22) and 37- to 40-months (*n* = 13) were included in the study.

According to the ECBQ development paper (Putnam et al., 2006), this measure demonstrated moderate interrater reliability, longitudinal stability at a moderate to large level from the ages of six to 36 months, and adequate internal consistency. Regarding construct validity, studies have consistently found relations between ECBQ indicators and temperament scores obtained in infancy and childhood (Putnam et al., 2018), as well as behavior problems (Gartstein et al., 2012), including in countries other than the United States (Gonzalez-Salinas et al., 2018). A study examining the Japanese version of the ECBQ further demonstrated that the measure showed internal consistency across its 18 scales and remained consistent across time (i.e., 18–36 months; Sukigara et al., 2015).

Over 20 papers document effective cross-cultural use of the ECBQ in the past 5 years, relating toddler temperament to constructs ranging from personality variables (Putnam and Gartstein, 2017) to parenting techniques (e.g., overprotective parenting; Jones et al., 2021) to developmental disorders (e.g., autism spectrum disorder, Vlaeminck et al., 2020; ADHD/ODD, Sánchez-Pérez et al., 2020). For each culture in this study, internal consistency reliability for all scores was examined, and items were subsequently dropped one-by-one across cultures to maximize the number of scales with *α* > 0.60 (Putnam et al., 2018). As a result, three items were eliminated from activity level, two were deleted from both attention focusing and impulsivity, and one item each was removed from attention shifting, low-intensity pleasure, and shyness. These deletions did not disrupt the content balance of the scale. Though internal consistency reliability for impulsivity remained below 0.60 in eight countries and did not improve with item deletion, the items resulting in the most optimal internal consistency were utilized to compute the Surgency overarching score. Overarching domain scores had good internal consistency reliability across all 14 countries (Desmarais et al., 2021a, b).

The Daily Activities Questionnaire (DAQ; Gartstein and Putnam, 2018), a parent-report questionnaire designed to ascertain how often parents of toddlers currently engaged in caregiving practices and other behaviors intended to maintain the household and support child-rearing was used to measure various aspects of daily routine, including sleep-supporting parenting techniques. The DAQ is composed of 46 items, rated on a 6-point rating scale with responses that range from “0-Never” to “5-Very Often.” For the purpose of our study, we examined the section of the DAQ that asked about parental techniques used to assist children in falling asleep. Based on an exploratory factor analysis, these techniques were further categorized into active sleep-supporting techniques (i.e., walking in the stroller, going for a car ride, walking while holding, doing a special play activity) and passive sleep-supporting techniques (i.e., talking softly, reading stories, cuddling, and singing), following a “data-driven” approach (for more details see Putnam et al., 2018). The resulting 4-item scale reflecting active sleep techniques generated alphas > 0.60 in 9 of 14 countries, and the 4-item passive sleep techniques scale alphas were > 0.60 in 6 countries. The DAQ was developed for use by the parent JETTC project (Gartstein and Putnam, 2018), with preliminary analyses supporting cross-cultural applicability of this instrument (Kirchhoff et al., 2014). The measure has also been used in other studies examining child temperament (Huitron et al., 2017), television exposure and behavioral/emotional dysregulation (Desmarais et al., 2021a), and mothers’ socialization goals and ethnotheories (Majdandzic et al., 2017), based on the parent JETTC project.

### Analytic strategy

We utilized a linear MLM approach to examine between-and within-cultural differences in parental sleep techniques (i.e., active and passive) in relation to toddler temperament. Child age and gender were included as covariates because they have been previously linked to temperament (Gartstein and Rothbart, 2003; Else-Quest et al., 2006; Putnam et al., 2006; Casalin et al., 2012) and to maintain consistency with prior cross-cultural studies (e.g., Montirosso et al., 2011; Cozzi et al., 2013; Slobodskaya et al., 2013). Data for active and passive sleep practices were group-mean centered, meaning that the arithmetic mean rating for sleep practices in each culture was subtracted from the individual ratings of all subjects within a culture for both sleep scales (Enders and Tofighi, 2007). This procedure allows for assessment of both between-and within-group effects. That is, the mean for each culture (i.e., level 2 variables) and individual-level group-mean centered values (i.e., level 1 variables) were included in all models to assess not only the culture-level effect of sleep practices (represented by the cultural mean), but also the effects of differing from normative practices within one’s culture (represented by group-mean centered values). Although parents with the same cultural backgrounds vary somewhat with respect to sleep-related practices, there are also strong culture-wide prescriptions regarding sleep for young children, which caregivers typically follow closely. Thus, we sought to understand the unique influence of both normative cultural practices and individual differences within culture.

Models were constructed in three phases, starting with a Null Model that partitioned within-and between-level variance and provided an unconditional intraclass correlation coefficient estimate for comparing subsequent models. Model 1 added age and gender covariates. The Final Model introduced group-mean centered sleep practices (i.e., level 1 variables) as well as group-mean values (i.e., level 2 variables) in order to account for within- and between-culture variance, respectively. This final model can be noted as


(1) $$\begin{array}{c} {\mathrm{Temperament}}_{\mathrm{ij}}=\gamma_{00}+\gamma_{01}\left({\mathrm{Age}}_{\mathrm{ij}}\right)+\gamma_{02}\left({\mathrm{Gender}}_{\mathrm{ij}}\right)+\\ \gamma_{03}\left({\mathrm{Active}}_{i-j}\right)++\gamma_{04}\left({\mathrm{Passive}}_{i-j}\right)+\\ \gamma_{10}\left({\mathrm{Active}}_{j}\right)+\gamma_{20}\left({\mathrm{Passive}}_{j}\right)+\mu_{0j}+r_{ij}. \end{array}$$

where Temperament_ij_ represents an individual’s rating on a specific temperament variable, *γ*_01_(Age_ij_) indicates the coefficient associated with a subject’s age in months, *γ*_02_ (Gender*_ij_*) indicates the coefficient associated with a subject’s gender. The parameters *γ*_03_ (Active_*i*–*j*_) and γ_04_ (Passive_*i*–*j*_) indicate the coefficients associated with the difference between the culture-mean and subject’s reported use of active or passive techniques (i.e., level 1/individual-level variables), respectively, and *γ*_10_ (Active*_j_*) and *γ*_20_ (Passive*_j_*) indicate the coefficients associated with the culture-level mean (i.e., group mean) for active and passive techniques, respectively.

Significant effects for cultural means (i.e., represented by both *γ*_10_ and *γ*_20_) indicate that the average frequency of use of a specific parental sleeping technique (i.e., active or passive) within a culture predicts individual differences in temperament. Significant group-mean centered effects (i.e., represented by *γ*_03_ and *γ*_04_) indicate that the degree to which an individual differs from the cultural average accounts for variance in temperament.

Models were compared *via* various fit indices [i.e., Akaike Information Criterion (AIC), Bayesian Information Criterion (BIC), Chi-square]. Models were estimated using restricted maximum likelihood (REML) to accommodate the relatively low number of level-2 groups (i.e., cultures, *J* = 14). Models were also estimated using full information maximum likelihood for the purposes of the chi-square difference test based on the deviance statistic. Models were also assessed in terms of variance accounted for by sleep practices. The intraclass correlation (ICC) reflects the proportion of variance occurring at the culture-level in comparison to the total model variance. Similarly, models were also compared based upon reduction of between-and within-culture variance explained by sleep practices in comparison to models with only age and gender covariates utilizing equation 1 as described by Hox et al. (2017):


(2) $$\begin{array}{c} \Delta R^{2}=\left(\mathrm{Model}\;1\;\mathrm{Estimate}–\mathrm{Final Model Estimate}\right)/\\ \left(\mathrm{Model}\;1\;\mathrm{Estimate}\right). \end{array}$$

Importantly, Δ*R*^2^ reflects the relative (i.e., proportional) difference in between- or within-level variance statistic. That is, we can look at change in each level of variance. Thus, the change in *R*^2^ values discussed herein reflect the percentage reduction in between-and within-culture variance when adding sleep practice variables to the previous model, which included only age and gender covariates.

## Results

Table 2 provides summary statistics for final models, including changes in ICC, variance accounted for, and coefficients and standard effect sizes for individual- and culture-level sleep technique variables. Importantly, effect sizes are interpreted in the metric of the standard deviation and the term “effect” is used in the statistical sense of the word, not to imply causality. For example, a one standard deviation increase in the group-mean of passive sleep practices was associated with a 0.417 unit decrease in ratings of temperament discomfort. All models demonstrated better fit with regard to change in AIC, BIC, and chi-square deviance statistics (*χ*^2^ > 9.49, *p* < 0.05). Detailed models including covariates are presented in the supplementary results (Supplementary Tables 1–20), however, as the effect of age and gender were not a focus of this study, they will not be further discussed.

**Table 2:** Summary statistics for final models.

Factor/Scale	Null ICC	Model 1 ICC	Final Model ICC	Δ*R*^2^ Between^1^	Δ*R*^2^ Within^2^	Passive (Individual)		Active (Individual)		Passive (Culture)		Active (Culture)	
	(%)	(%)	(%)	(%)	(%)	*γ*	*δ*	*γ*	*δ*	*γ*	*δ*	*γ*	*δ*
Surgency	8.09	7.93	5.73	26.09	0.00	0.035	0.049	0.047	0.063	0.295	0.180	0.058	0.034
Activity level	1.69	1.92	2.48	0.00	0.00	0.008	0.007	0.104	0.087	−0.016	−0.006	−0.080	−0.029
High-intensity pleasure	13.20	13.11	8.28	39.80	0.00	0.024	0.021	0.060	0.050	−0.130	−0.050	0.659	0.242
Positive anticipation	10.44	10.21	9.72	4.48	0.00	0.099	0.091	0.017	0.015	−0.118	−0.047	0.354	0.137
Sociability	11.11	11.41	4.50	63.27	0.00	0.071	0.058	0.059	0.046	0.752^***^	0.267	0.196	0.067
Negative emotionality	20.88	20.46	8.92	62.07	0.88	−0.011	−0.016	0.107^***^	0.145	−0.568^***^	−0.351	0.228	0.136
Discomfort	28.30	27.69	9.77	72.02	1.23	0.039	0.033	0.174^***^	0.139	−1.149^***^	−0.417	0.477	0.167
Fear	19.12	18.89	8.31	60.61	0.00	−0.009	−0.008	0.093	0.081	−0.811^***^	−0.320	0.427	0.162
Frustration	8.06	7.99	8.91	0.00	0.00	0.019	0.017	0.122	0.105	0.088	0.034	0.157	0.059
Sadness	6.98	6.81	4.23	40.43	0.00	−0.043	−0.039	0.140	0.123	−0.454	−0.181	−0.002	−0.001
Shyness	3.99	3.92	3.13	20.59	0.00	−0.006	−0.005	0.012	0.009	−0.347	−0.123	−0.017	−0.006
Motor activity	11.53	12.88	11.34	14.52	0.95	−0.022	−0.024	0.145^***^	0.151	−0.339	−0.161	0.235	0.107
Perceptual sensitivity	13.45	12.72	6.03	56.20	0.11	0.165^***^	0.119	0.086	0.059	−0.833^***^	−0.262	0.348	0.105
Soothability	12.14	11.79	5.80	54.22	0.00	0.057	0.051	−0.088	−0.076	0.683^***^	0.267	−0.193	−0.073
Effortful control	3.23	3.45	2.51	33.33	1.22	0.123^***^	0.177	0.001	0.001	0.094	0.059	0.161	0.097
Attention focusing	1.67	1.37	0.46	33.33	0.00	0.065	0.058	−0.014	−0.012	−0.260	−0.100	0.098	0.036
Attention shifting	6.53	6.42	3.95	42.31	0.00	0.050	0.059	0.039	0.044	0.016	0.008	0.341	0.168
Cuddliness	8.03	8.06	7.04	13.27	0.00	0.146^***^	0.137	−0.023	−0.021	0.289	0.118	0.203	0.080
Inhibition	7.89	8.58	9.95	0.00	0.00	0.124	0.101	−0.027	−0.021	0.006	0.002	0.109	0.037
Low-intensity pleasure	6.74	6.94	4.43	40.00	4.69	0.234^***^	0.234	0.028	0.027	0.411	0.178	0.106	0.044

1 Between-culture variance (Δ*R*^2^ Between) reflects reduction in between-level variance attributed to sleep practices while controlling for age and gender covariates.

2 Within-culture variance (Δ*R*^2^ Within) reflects reduction in within-level variance attributed to sleep practices while controlling for age and gender covariates.

*γ*, unstandardized coefficient; *δ*, standardized coefficient; and ICC, interclass correlation.

*** *p* < 0.001.

“Model 1” reflects ICC for models with age and gender covariates.

“Model 2” reflects the ICC after including sleep practices.

The ICC and change in *R*^2^ are the most common metrics for comparing models in terms of variance and practical significance. Reductions in the ICC represent a decrease in the ratio of between- to within-culture variance. The interpretation of *R*^2^ is more nuanced in that it differs in MLM relative to standard multiple regressions. In MLM, *R*^2^ reflects the relative (i.e., proportional) difference in variance statistic between models. Thus, the change in *R*^2^ values presented in Table 2 reflect the percentage reduction in between- and within-culture variance when adding sleep practice variables to the previous model, which included only age and gender covariates.

For example, the Null Model ICC for models assessing NE was ~ 20.88%, meaning that ~ 20.88% of the total variance in NE occurred at the cultural level. In other words, if two random individuals were sampled from a given culture, we expect their NE scores to be correlated at 0.21. Adding age and gender covariates reduced the ICC to ~ 20.46%. The addition of sleep practice variables resulted in an ICC of ~ 8.92%, meaning only ~ 8.92% of all the remaining variance in NE occurred at the cultural level after accounting for the effects of sleep practices. In terms of change in *R*^2^, the addition of sleep practice variables explained ~ 62.07% of the between-culture variance and ~ 0.88% of the within-culture variance remaining after controlling for the effects of age and gender. In other words, after accounting for age and gender covariates, over half of the remaining variance in culture-level ratings of NE was explained by sleep practice variables. In contrast, very little individual-level variance in NE ratings was explained by sleep practice variables after accounting for age and gender. All other models summarized in Table 2 can be interpreted in the same manner.

Given the multiple statistical significance tests, the Benjamini–Hochberg procedure (Benjamini and Hochberg, 1995) to control the false discovery rate was employed and a conservative *p* > 0.001 was utilized to assess significance. Statistically significant results are presented in the text and Table 2. Greater use of passive sleep practices at the cultural level were significantly associated with higher sociability and soothability, and lower NE, discomfort, fear, and perceptual sensitivity. Effects for culture-level active sleep practices did not reach significance (*p* > 0.001). Regarding the effects of individual variations within cultures (i.e., deviations from the group mean predicting changes in the individual temperament ratings), passive sleep practices were positively associated with EC, perceptual sensitivity, cuddliness, and low-intensity pleasure. At the individual level, active sleep techniques were positively associated with NE, discomfort, and motor activity.

Rank-ordering the extent to which a culture’s sample endorsed using passive techniques (Supplementary Figure 1), we find that the United States, Finland, and Netherlands top the list and South Korea, Turkey, and China are at the bottom of this distribution. In contrast, rank-ordering for active techniques (Supplementary Figure 2), we find that Romania, Spain, and Chile top the list while Turkey, Italy, and Belgium are at the bottom of the distribution.

## Discussion

The present study examined parental sleep-supporting practices during toddlerhood in relation to temperament across 14 cultures. Overall, the addition of sleep practice variables to our null models explained from 0.00–72.02% of between-culture temperament variance and 0.00–4.69% of within-culture temperament variance, after controlling for the effects of age and gender. Thus, sleep practices appeared to account for variance more consistently at the between-culture level, and these effects were generally proportionally larger than the ones that emerged at the within-culture level. The size of between-culture effects suggests that parental sleep-supporting practices make substantial contributions to cross-cultural differences in child temperament. Overall, passive sleep-supporting techniques (e.g., cuddling) were associated with temperament outcomes at the culture level (e.g., higher levels of sociability, lower NE) and at the individual level (e.g., higher levels of EC), whereas active sleep-supporting techniques (e.g., doing an activity together) were associated with temperament outcomes at an individual level only (e.g., higher NE), largely supporting hypotheses.

Culture-level associations between passive sleep-supporting techniques and temperament are consistent with previous findings indicating countries where parents reported frequent reliance on passive techniques also had toddlers with higher levels of SUR and lower levels of NE (Gartstein and Putnam, 2018). Results in Supplementary Figure 1 demonstrate that cultures categorized as “individualistic,” or more Western in their orientation, rather than “collectivistic” tend to use more passive approaches to soothe their child. These results appear to be in line with those reported by Sadeh et al. (2011) who found that parents from predominantly Caucasian (PC) cultures were less likely than those from predominantly Asian (PA) cultures to describe their children as struggling with sleep issues (linked with more active sleep-supporting techniques, e.g., Morrell and Cortina-Borja, 2002). Prior studies demonstrating a combination of fewer child sleep issues (Sadeh et al., 2011) and a tendency toward less involved parenting behaviors related to sleep (e.g., waiting for the child to independently fall asleep; Mindell et al., 2010) can be viewed as consistent with the present findings suggesting that parents in countries with frequent endorsement of passive sleep-inducing techniques report lower NE in their children and higher positive affectivity. However, results for usage of active techniques per culture (Supplementary Figure 2) do not show a consistent pattern based on a cultural endorsement of individualism. This pattern of results seems to indicate that not only are active sleep-supporting techniques used less frequently by parents relative to passive ones overall, but that there may be less of a cultural effect on the active set of sleep-supporting behaviors.

The substantial variability in the percentage of each temperament dimension accounted for by sleep practices at a between-culture level could reflect differences in cultural values/priorities. It may also be that other factors (e.g., customary bedtime and sleep beliefs, presence of other relatives, physical sleep arrangements, electronic device usage before bedtime) influenced by culture take precedence over parental sleep-related interventions for some manifestations of temperament but not others—possibilities that should be considered in future cross-cultural investigations. Cultural norms regarding how much parents attend to child sleep patterns have been linked with caregivers’ appraisals of other areas of child functioning, including temperament (Jenni and O’Connor, 2005; Giannotti and Cortesi, 2009; Mindell et al., 2010), and may be differentially related to various attributes.

Higher proportions of variance accounted for by parenting practices across fine-grained dimensions and overall NE suggest that this contextual factor (i.e., parental sleep-supporting practices) has stronger connections with distress proneness relative to SUR or EC at the between culture level, that is, in terms of distinguishing among cultures rather than individuals. In contrast to other dimensions of NE and the overarching factor itself, soothability was positively related to passive techniques, which is not surprising given that this scale loads negatively onto the NE factor. Passive sleep induction techniques likely assist infants in developing self-soothing and regulation (Öztürk Dönmez and Bayik Temel, 2019), in turn leading to greater soothability in non-sleep contexts. At the fine-grained level, discomfort, fear, perceptual sensitivity and soothability demonstrated the strongest relations with respect to between culture effects, thus may be more closely linked with cultural differences in sleep relative to other aspects of NE.

Although significant results were not observed for overall SUR, there was a significant between culture effect for passive sleep induction techniques and sociability—countries with greater reliance on passive strategies had toddlers with higher sociability scores. Sociability may be unique among members of the SUR constellation, with greater cross-cultural variability related to sleep and parental approach to supporting sleep in toddlers. This may be due to its role in the development of social competence, which has been associated with sleep consolidation (Mindell et al., 2017), duration, and onset (Tomisaki et al., 2018) in infancy and toddlerhood.

There is a considerable amount of research examining the association between parent sleep-soothing techniques and child sleep difficulties (at the individual, but not cultural level) as well as linking temperament to sleep difficulties, yet limited efforts have addressed the association between parent sleep-soothing techniques and child temperament. A previous study found that fussy-difficult temperament in 14–16-month-old infants was positively correlated with physical comforting—characterized by cuddling or settling in the parent’s bed, rocking in the parent’s arms, or giving food/drink to assist with settling the child to sleep (Morrell and Steele, 2003). Similarly, parents with temperamentally difficult 12- to 19-month-old children used more physical comforting strategies (e.g., cuddling, rocking, giving them food/drink) than parents with temperamentally easy children (Morrell and Cortina-Borja, 2002). Earlier measures were less differentiated than the assessment tools used in this study, and our results extend prior findings by suggesting that active techniques that involve removing the child from bed to walk, drive or play with them are related specifically to greater distress proneness. This extension further supports the idea that clinicians suggest passive sleep-supporting techniques to parents to interrupt the pattern of active techniques perpetuating temperament-related sleep difficulties.

Negative emotionality, which operationally overlaps with fussy-difficult temperament examined in previous studies, was significantly correlated with active sleep techniques but not passive strategies on an individual level. A previous study investigating the relationship between parents’ comforting techniques and child sleep behavior indicated that mothers who used active strategies (e.g., rocking, rubbing the child’s back) reported problematic child sleep patterns (the child had to be comforted/resettled) and frequent nighttime waking in preschoolers (Coulombe and Reid, 2014). Sleep disturbances (e.g., delayed sleep onset, nightmares, and restless sleep) were positively correlated with children’s (mean age 5.7 years) temperamental emotionality, conceptually similar to the NE factor on the ECBQ (Owens-Stively et al., 1997). Furthermore, Ward et al. (2008) reported temperament differences in preschoolers based on napping behavior. Those who were “problem nappers” (e.g., children who struggled to settle down or exhibited disruptive behavior) had lower effortful control (EC) and higher NE scores. Overall, this pattern of results indicates that active sleep-supporting parenting strategies are associated with greater child NE, consistently linked with sleep difficulties in existing studies. Mindell and Williamson’s, 2018 recent review on cross-cultural prevalence of bedtime behaviors has pointed out that some aspects of previously adaptive behaviors may become non-adaptive with development, also varying in effectiveness depending on the child (e.g., “adaptive” singing being too overstimulating for some children). Thus, it may be that active sleep supporting practices interfere with sleep quality particularly for children with higher NE but not others. On a related note, child temperament may exert some influence on parental sleep-supporting behaviors, so that child NE contributes to active techniques, perhaps eliciting stimulating responses from caregivers starting in infancy. Future research should examine this direction of effects, also considering the role of sleep problems linked to emotional/behavioral problems and to higher NE in this context (e.g., emotional reactivity as a risk factor for sleep problems, Baukienė and Jusienė, 2021; infants of Caesarean deliveries having elevated sleep problems as well as internalizing difficulties, Kelmanson, 2003). Future research should extend the present investigation by also considering sleep difficulties across cultures, utilizing actigraphy along with parent-report to examine difficulty patterns that may be specific to culture and better inform sleep-targeted interventions.

It should be noted that the NE dimension of perceptual sensitivity was positively associated with passive soothing techniques, whereas positive associations for discomfort and motor activation were observed with active strategies. Perceptual sensitivity involves children’s ability to flexibly participate in quiet activities and toddlers’ awareness of mild, low-intensity stimuli (Putnam et al., 2006), which may explain its links to passive sleep-soothing techniques, which tend to be quiet, gentle, and less stimulating. More active techniques were associated with greater discomfort and motor activation within cultures, in line with between-culture results indicating passive techniques tend to be conducive to lower NE overall.

Overall EC as well as fine-grained dimensions of cuddliness and low-intensity pleasure were positively related to passive techniques at an individual level. As passive techniques consist of talking softly, reading stories, cuddling, and singing, they may directly promote behavioral manifestations of these narrowly defined attributes (i.e., enjoying closeness and activities offering less complexity and stimulation), explaining the overall EC within-culture effect. It should be noted that smaller amounts of within-culture temperament variance (0.00–4.69%) accounted for with the addition of sleep variables to our null models could be indicative of other factors contributing to individual differences. This pattern of results may reflect relative importance of other contextual factors within cultures, for example overall quality of caregiving (e.g., sensitivity/responsiveness; Gartstein et al., 2008; Leclère et al., 2014), which should be examined in future research.

This study has several limitations. First, internal consistency of the active and passive sleep techniques measure was lower than optimal in several cultures. Utility of the DAQ is evident given a number of hypothesized effects that emerged herein; however, this measure will benefit from further study and possible refinement. For example, future research should consider if DAQ sleep-supporting techniques scales account for variance in temperament outcomes similar to more comprehensive and lengthy instruments such as the Parental Interactive Bedtime Behavior Scale (PIBBS; Morrell and Cortina-Borja, 2002). A second limitation of the study results from the DAQ and ECBQ being parent-report questionnaires. In future research, observational measure of temperament and sleep-supporting techniques should be considered to increase the confidence in the pattern of results observe herein. A third limitation has to do with the cross-sectional nature of the study, which does not permit us to make causal interpretations. Longitudinal investigations are needed to discern whether infants with more challenging temperament profiles (i.e., higher NE) elicit more active sleep-supporting techniques from the caregivers and to consider bi-directional effects. These studies should also track sleep problems discerning potential effects with respect to NE, as well as sleep-supporting parenting behaviors. Finally, though 14 cultures were compared in this study, this is a relatively small number and is limiting in terms of power using MLM. Future work examining the relationship between sleep practices and temperament outcomes should aim to collect data from a larger number of cultures to increase statistical power and afford further generalizability.

This study addresses the gap in the developmental sleep literature by exploring cross-cultural differences in the effects of sleep-supporting techniques on toddler temperament across 14 cultures. By examining associations from the overall temperament factor level and the fine-grained dimension level, this study links parental sleep-supporting techniques with specific dimensions that have been connected to developmental outcomes such as adjustment problems (e.g., low fear exacerbating maladjustment to stress for preschool-age children in high-risk contexts; Moran et al., 2017). Our findings indicate that both within-and between-culture differences in passive sleep-supporting techniques are associated with temperament attributes, and within-culture active techniques effects were also noted. Overall results highlight the importance of links between parental sleep practices and early temperament development, indicating that passive techniques are associated with more adaptive temperament profiles (e.g., lower NE, higher levels of sociability, and higher levels of EC). Notably, a greater amount of between-culture level variance was explained relative to the within-culture level. Implications include potentially targeting sleep-related parenting practices to support temperament development, facilitating positive adjustment/behavioral health across cultures. Future research will need to further support current findings and examine potential benefits of such applications, extending the present investigation.

## Data availability statement

The raw data supporting the conclusions of this article will be made available by the authors upon reasonable request.

## Ethics statement

The studies involving human participants were reviewed and approved by Washington State University Institutional Review Boards. The patients/participants provided their written informed consent to participate in this study.

## Author contributions

MG and SP contributed to the conception and design of the study. ZW, SP, SC, ML, FL, ST, KH, KR, RM, LG, S-YP, S-YH, EL, BH, CW, RB, MM, CG-S, IA, HS, EK, EA, OB, and MG collected the data and managed sites in each respective location. ED and BF performed the statistical analyses. CP wrote the first draft of the manuscript. CP, ED, and VJ wrote sections of the manuscript. All authors contributed to the article and approved the submitted version.

## Funding

This project was supported by Washington State University College of Arts and Sciences Berry Family Faculty Excellence Award awarded to MG.

## Conflict of interest

The authors declare that the research was conducted in the absence of any commercial or financial relationships that could be construed as a potential conflict of interest.

## Publisher’s note

All claims expressed in this article are solely those of the authors and do not necessarily represent those of their affiliated organizations, or those of the publisher, the editors and the reviewers. Any product that may be evaluated in this article, or claim that may be made by its manufacturer, is not guaranteed or endorsed by the publisher.

## Supplementary material

The Supplementary material for this article can be found online at: https://www.frontiersin.org/articles/10.3389/fpsyg.2022.1004082/full#supplementary-material
