# Supplementary material for: Relations between bedtime parenting behaviors and temperament across 14 cultures
Source: Front Psychol. 2022 Nov 24;13:1004082. doi: 10.3389/fpsyg.2022.1004082 (PMC9731114; doi:10.3389/fpsyg.2022.1004082)
Supplement: Supplementary file 1 [file Data_Sheet_1.pdf]

Figure 1. Passive Technique Usage: Rank-order by Culture

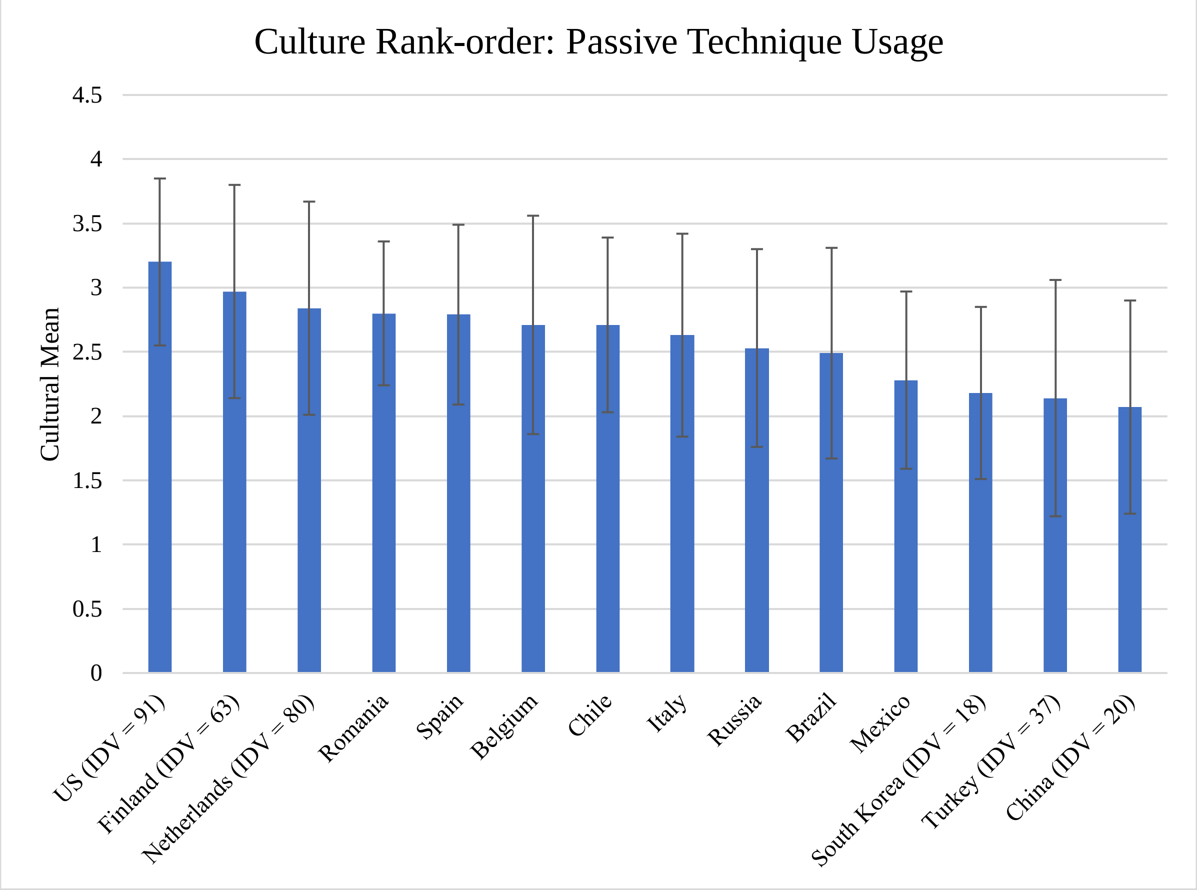

Notes: *N* (total number of cases) = 841, *J* (total number of cultures) = 14; Bars = Mean, Error bars = Standard deviation; IDV = Hofstede's cultural dimension of Individualism/Collectivism, higher value = more individualistic.

Figure 2. Active Technique Usage: Rank-order by Culture

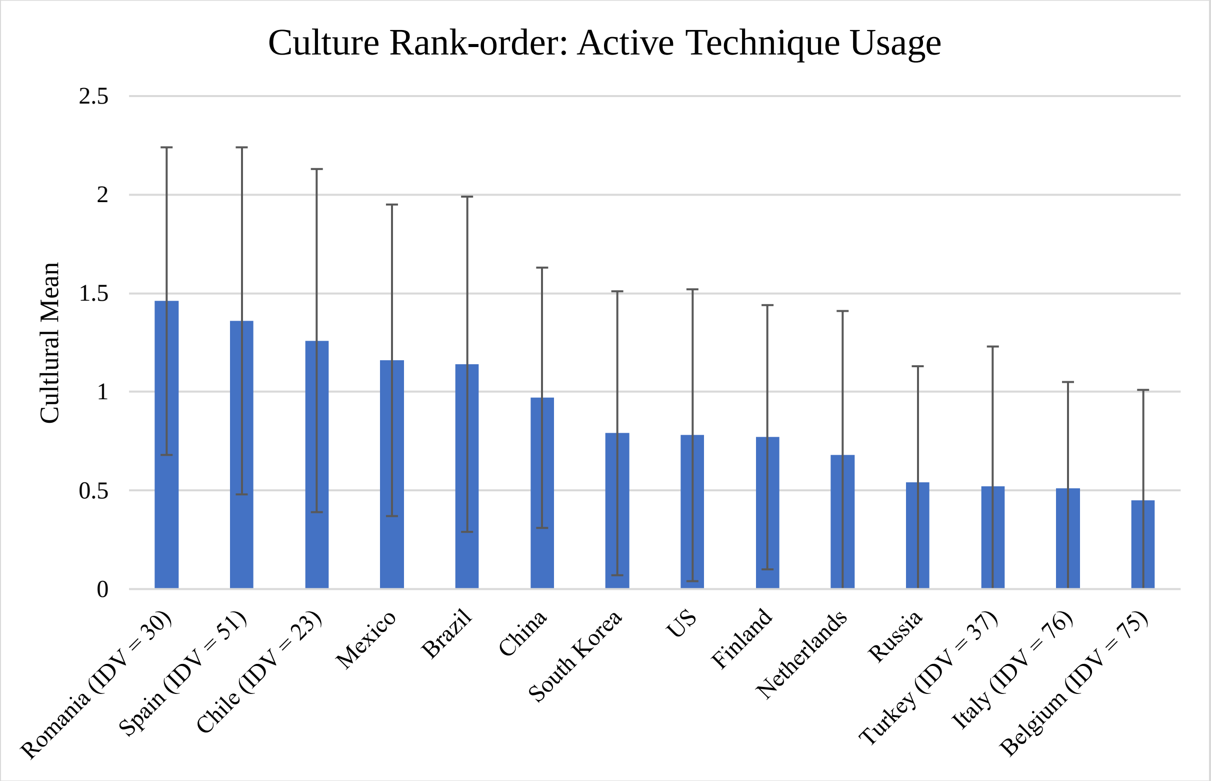

Notes: *N* (total number of cases) = 841, *J* (total number of cultures) = 14; Bars = Mean, Error bars = Standard deviation; IDV = Hofstede's cultural dimension of Individualism/Collectivism, higher value = more individualistic.

Table 1. Model estimates for ECBQ Surgency.

	Model 1		Model 2	
**Fixed Components**	Est.	*SE*	Est.	*SE*
Intercept	4.647	.110	3.801	.346
Age	.004	.003	.004	.003
Gender	.098**	.035	.110**	.036
Group-Mean Centered Gentle			.035	.025
Group-Mean Centered Active			.047	.026
Group Mean Gentle			.295*	.121
Group Mean Active			.058	.120
**Variance Components**				
**Within**	.269	.013	.272	.013
**Between**				
Intercept	.023	.011	.017	.010
Slope				
**Model Fit**				
χ^{2a}	1342.84		1307.02	
AIC	1371.78		1360.37	
BIC	1395.60		1402.98	
R^2 within (%)[b]			.00	
R^2 between (%)[b]			26.09	

Notes: Est = Estimate. *SE* = Standard Error

[a]χ^2 were estimated using full maximum likelihood.

[b] R^2 represents in the variance explained in comparison to model 1.

**p*<.05, ***p*<.01.

Table 2. Model estimates for ECBQ Activity Level.

	Model 1		Model 2	
**Fixed Components**	Est.	*SE*	Est.	*SE*
Intercept	4.637	.170	4.701	.436
Age	-.016**	.006	-.015***	.005
Gender	.200**	.058	.223**	.059
Group-Mean Centered Gentle			.008	.041
Group-Mean Centered Active			.104*	.043
Group Mean Gentle			-.016	.146
Group Mean Active			-.080	.146
**Variance Components**				
**Within**	.726	.035	.733	.036
**Between**				
Intercept	.014	.011	.019	.014
Slope				
**Model Fit**				
χ^{2a}	2185.83		2131.31	
AIC	2212.86		2179.59	
BIC	2236.68		2222.20	
R^2 within (%)[b]			.00	
R^2 between (%)[b]			.00	

Notes: Est = Estimate. *SE* = Standard Error

[a]χ^2 were estimated using full maximum likelihood.

[b] R^2 represents in the variance explained in comparison to model 1.

**p*<.05, ***p*<.01, ****p*<.001.

Table 3. Model estimates for ECBQ High-Intensity Pleasure.

	Model 1		Model 2	
**Fixed Components**	Est.	*SE*	Est.	*SE*
Intercept	4.408	.179	2.772	.623
Age	-.007	.005	-.006	.005
Gender	.203***	.055	.212***	.056
Group-Mean Centered Gentle			.024	.038
Group-Mean Centered Active			.060	.041
Group Mean Gentle			-.130	.219
Group Mean Active			.659**	.221
**Variance Components**				
**Within**	.646	.031	.658	.032
**Between**				
Intercept	.098	.043	.059	.030
Slope				
**Model Fit**				
χ^{2a}	2106.72		2052.93	
AIC	2132.55		2099.148	
BIC	2156.36		2141.759	
R^2 within (%)[b]			.00	
R^2 between (%)[b]			39.80	

Notes: Est = Estimate. *SE* = Standard Error

[a]χ^2 were estimated using full maximum likelihood.

[b] R^2 represents in the variance explained in comparison to model 1.

***p<.01, ***p<.001.

Table 4. Model estimates for ECBQ Anticipation.

	Model 1		Model 2	
**Fixed Components**	Est.	*SE*	Est.	*SE*
Intercept	4.352	.165	4.363	.634
Age	.028***	.004	.026***	.005
Gender	.022	.052	.032	.053
Group-Mean Centered Gentle			.099**	.037
Group-Mean Centered Active			.017	.039
Group Mean Gentle			-.118	.226
Group Mean Active			.354	.224
**Variance Components**				
**Within**	.586	.029	.593	.029
**Between**				
Intercept	.067	.030	.064	.032
Slope				
**Model Fit**				
χ^{2a}	2017.50		1971.19	
AIC	2043.88		2014.64	
BIC	2067.69		2057.25	
R^2 within (%)[b]			.00	
R^2 between (%)[b]			4.48	

Notes: Est = Estimate. *SE* = Standard Error

[a]χ^2 were estimated using full maximum likelihood.

[b] R^2 represents in the variance explained in comparison to model 1.

**p<.01, ***p<.001.

Table 5. Model estimates for ECBQ Sociability.

	Model 1		Model 2	
**Fixed Components**	Est.	*SE*	Est.	*SE*
Intercept	4.950	.191	2.793	.535
Age	.021***	.005	.021***	.005
Gender	-.031	.060	-.017	.061
Group-Mean Centered Gentle			.071	.042
Group-Mean Centered Active			.059	.044
Group Mean Gentle			.752***	.185
Group Mean Active			.196	.184
**Variance Components**				
**Within**	.763	.037	.774	.038
**Between**				
Intercept	.098	.044	.036	.021
Slope				
**Model Fit**				
χ^{2a}	2248.49		2182.86	
AIC	2273.97		2229.49	
BIC	2297.78		2272.10	
R^2 within (%)[b]			.00	
R^2 between (%)[b]			63.27	

Notes: Est = Estimate. *SE* = Standard Error
[a]χ^2 were estimated using full maximum likelihood.
[b] R^2 represents in the variance explained in comparison to model 1.
****p*<.001.

Table 6. Model estimates for ECBQ Negative Emotionality.

	Model 1		Model 2	
**Fixed Components**	Est.	*SE*	Est.	*SE*
Intercept	2.897	.114	4.109	.376
Age	.007*	.003	.008**	.003
Gender	-.042	.033	-.033	.033
Group-Mean Centered Gentle			-.011	.023
Group-Mean Centered Active			.107***	.024
Group Mean Gentle			-.568***	.134
Group Mean Active			.228	.132
**Variance Components**				
**Within**	.227	.011	.225	.011
**Between**				
Intercept	.058	.024	.022	.011
Slope				
**Model Fit**				
χ^{2a}	1206.48		1151.28	
AIC	1234.97		1204.81	
BIC	1258.78		1247.42	
R^2 within (%)[b]			.88	
R^2 between (%)[b]			62.07	

Notes: Est = Estimate. *SE* = Standard Error
[a]χ^2 were estimated using full maximum likelihood.
[b] R^2 represents in the variance explained in comparison to model 1.
**p*<.05, ***p*<.01, ****p*<.001.

Table 7. Model estimates for ECBQ Discomfort.

	Model 1		Model 2	
**Fixed Components**	Est.	*SE*	Est.	*SE*
Intercept	2.031	.194	4.506	.619
Age	.025***	.005	.027***	.005
Gender	-.061	.052	-.049	.052
Group-Mean Centered Gentle			.039	.036
Group-Mean Centered Active			.174***	.038
Group Mean Gentle			-1.149***	.221
Group Mean Active			.477*	.219
**Variance Components**				
**Within**	.569	.028	.562	.028
**Between**				
Intercept	.218	.089	.061	.030
Slope				
**Model Fit**				
χ^{2a}	2007.96		1922.91	
AIC	2033.30		1969.76	
BIC	2057.11		2012.37	
R^2 within (%)[b]			1.23	
R^2 between (%)[b]			72.02	

Notes: Est = Estimate. *SE* = Standard Error

[a]χ^2 were estimated using full maximum likelihood.

[b] R^2 represents in the variance explained in comparison to model 1.

**p*<.05, ****p*<.001.

Table 8. Model estimates for ECBQ Fear.

	Model 1		Model 2	
**Fixed Components**	Est.	*SE*	Est.	*SE*
Intercept	2.459	.177	4.144	.582
Age	.014**	.005	.015**	.005
Gender	-.172**	.051	-.168**	.052
Group-Mean Centered Gentle			-.009	.036
Group-Mean Centered Active			.093*	.038
Group Mean Gentle			-.811***	.207
Group Mean Active			.427*	.204
**Variance Components**				
**Within**	.565	.027	.572	.028
**Between**				
Intercept	.132	.056	.052	.026
Slope				
**Model Fit**				
χ^{2a}	1995.70		1935.27	
AIC	2021.53		1982.46	
BIC	2045.35		2025.07	
R^2 within (%)[b]			.00	
R^2 between (%)[b]			60.61	

Notes: Est = Estimate. *SE* = Standard Error

[a] χ^2 were estimated using full maximum likelihood.

[b] R^2 represents in the variance explained in comparison to model 1.

**p*<.05, ***p*<.01, ****p*<.001.

Table 9. Model estimates for ECBQ Frustration.

	Model 1		Model 2	
**Fixed Components**	Est.	*SE*	Est.	*SE*
Intercept	3.337	.172	2.916	.646
Age	.002	.005	.003	.005
Gender	.062	.055	.075	.057
Group-Mean Centered Gentle			.019	.039
Group-Mean Centered Active			.122**	.041
Group Mean Gentle			.088	.230
Group Mean Active			.157	.227
**Variance Components**				
**Within**	.658	.032	.664	.033
**Between**				
Intercept	.057	.027	.065	.033
Slope				
**Model Fit**				
χ^{2a}	2114.77		2062.05	
AIC	2141.03		2108.00	
BIC	2164.85		2150.61	
R^2 within (%)[b]			.00	
R^2 between (%)[b]			.00	

Notes: Est = Estimate. *SE* = Standard Error

[a]χ^2 were estimated using full maximum likelihood.

[b] R^2 represents in the variance explained in comparison to model 1.

***p*<.01.

Table 10. Model estimates for ECBQ Sadness.

	Model 1		Model 2	
**Fixed Components**	Est.	*SE*	Est.	*SE*
Intercept	2.749	.167	3.850	.478
Age	.006	.005	.009	.005
Gender	.021	.055	.030	.056
Group-Mean Centered Gentle			-.043	.038
Group-Mean Centered Active			.140**	.040
Group Mean Gentle			-.454**	.165
Group Mean Active			-.002	.164
**Variance Components**				
**Within**	.637	.031	.644	.032
**Between**				
Intercept	.047	.023	.028	.017
Slope				
**Model Fit**				
χ^{2a}	2085.54		2027.70	
AIC	2112.04		2075.79	
BIC	2135.86		2118.40	
R^2 within (%)[b]			.00	
R^2 between (%)[b]			40.43	

Notes: Est = Estimate. *SE* = Standard Error

[a]χ^2 were estimated using full maximum likelihood.

[b] R^2 represents in the variance explained in comparison to model 1.

***p*<.01.

Table 11. Model estimates for ECBQ Shyness.

	Model 1		Model 2	
**Fixed Components**	Est.	*SE*	Est.	*SE*
Intercept	3.551	.184	4.455	.497
Age	-.005	.005	-.005	.006
Gender	-.150*	.062	-.148*	.064
Group-Mean Centered Gentle			-.006	.044
Group-Mean Centered Active			.012	.046
Group Mean Gentle			-.347*	.168
Group Mean Active			-.017	.168
**Variance Components**				
**Within**	.822	.040	.840	.041
**Between**				
Intercept	.034	.019	.027	.018
Slope				
**Model Fit**				
χ^{2a}	2299.61		2247.39	
AIC	2325.80		2294.24	
BIC	2349.61		2336.86	
R^2 within (%)[b]			.00	
R^2 between (%)[b]			20.59	

Notes: Est = Estimate. *SE* = Standard Error

[a]χ^2 were estimated using full maximum likelihood.

[b] R^2 represents in the variance explained in comparison to model 1.

*p<.05.

Table 12. Model estimates for ECBQ Motor Activity.

	Model 1		Model 2	
**Fixed Components**	Est.	*SE*	Est.	*SE*
Intercept	2.608	.144	3.195	.571
Age	-.019***	.004	-.017***	.004
Gender	.022	.044	.038	.045
Group-Mean Centered Gentle			-.022	.031
Group-Mean Centered Active			.145***	.032
Group Mean Gentle			-.339	.205
Group Mean Active			.235	.202
**Variance Components**				
**Within**	.421	.020	.417	.021
**Between**				
Intercept	.062	.027	.053	.026
Slope				
**Model Fit**				
χ^{2a}	1736.15		1674.53	
AIC	1763.28		1723.04	
BIC	1787.10		1765.65	
R^2 within (%)[b]			.95	
R^2 between (%)[b]			14.52	

Notes: Est = Estimate. *SE* = Standard Error

[a]χ^2 were estimated using full maximum likelihood.

[b] R^2 represents in the variance explained in comparison to model 1.

****p*<.001.

Table 13. Model estimates for ECBQ Perceptual Sensitivity.

	Model 1		Model 2	
**Fixed Components**	Est.	*SE*	Est.	*SE*
Intercept	3.907	.215	5.734	.655
Age	.021***	.006	.021***	.006
Gender	-.024	.066	-.009	.067
Group-Mean Centered Gentle			.165***	.047
Group-Mean Centered Active			.086	.049
Group Mean Gentle			-.833***	.348
Group Mean Active			.348	.228
**Variance Components**				
**Within**	.942	.046	.941	.046
**Between**				
Intercept	.137	.060	.060	.033
Slope				
**Model Fit**				
χ^{2a}	2431.66		2349.79	
AIC	2456.38		2394.36	
BIC	1480.20		2436.97	
R^2 within (%)[b]			.11	
R^2 between (%)[b]			56.20	

Notes: Est = Estimate. *SE* = Standard Error

[a]χ^2 were estimated using full maximum likelihood.

[b] R^2 represents in the variance explained in comparison to model 1.

****p*<.001.

Table 14. Model estimates for ECBQ Soothability.

	Model 1		Model 2	
**Fixed Components**	Est.	*SE*	Est.	*SE*
Intercept	5.495	.173	3.956	.527
Age	-.011*	.005	-.013*	.005
Gender	.034	.054	.032	.055
Group-Mean Centered Gentle			.057	.038
Group-Mean Centered Active			-.088*	.040
Group Mean Gentle			.683***	.184
Group Mean Active			-.193	.183
**Variance Components**				
**Within**	.620	.030	.626	.031
**Between**				
Intercept	.083	.036	.038	.021
Slope				
**Model Fit**				
χ^{2a}	2068.69		2006.18	
AIC	2094.76		2053.68	
BIC	2118.57		2096.29	
R^2 within (%)[b]			.00	
R^2 between (%)[b]			54.22	

Notes: Est = Estimate. *SE* = Standard Error

[a]χ^2 were estimated using full maximum likelihood.

[b] R^2 represents in the variance explained in comparison to model 1.

**p*<.05, ****p*<.001.

Table 15. Model estimates for ECBQ Effortful Control.

	Model 1		Model 2	
**Fixed Components**	Est.	*SE*	Est.	*SE*
Intercept	4.169	.100	3.806	.251
Age	.026***	.003	.025***	.003
Gender	-.120***	.034	-.117**	.034
Group-Mean Centered Gentle			.123***	.024
Group-Mean Centered Active			.001	.025
Group Mean Gentle			.094	.084
Group Mean Active			.161	.084
**Variance Components**				
**Within**	.245	.012	.242	.012
**Between**				
Intercept	.009	.005	.006	.005
Slope				
**Model Fit**				
χ^{2a}	1250.12		1197.80	
AIC	1280.03		1253.85	
BIC	1303.84		1296.47	
R^2 within (%)[b]			1.22	
R^2 between (%)[b]			33.33	

Notes: Est = Estimate. *SE* = Standard Error

[a]χ^2 were estimated using full maximum likelihood.

[b] R^2 represents in the variance explained in comparison to model 1.

**p<.01, ***p<.001.

Table 16. Model estimates for ECBQ Attention Focusing.

	Model 1		Model 2	
**Fixed Components**	Est.	*SE*	Est.	*SE*
Intercept	3.415	.161	4.094	.321
Age	.044***	.005	.043***	.005
Gender	-.056	.055	-.062	.057
Group-Mean Centered Gentle			.065	.039
Group-Mean Centered Active			-.014	.041
Group Mean Gentle			-.260**	.098
Group Mean Active			.043	.100
**Variance Components**				
**Within**	.660	.032	.673	.033
**Between**				
Intercept	.009	.008	.003	.006
Slope				
**Model Fit**				
χ^{2a}	2100.85		2050.00	
AIC	2128.33		2100.94	
BIC	2152.15		2143.55	
R^2 within (%)[b]			.00	
R^2 between (%)[b]			33.33	

Notes: Est = Estimate. *SE* = Standard Error

[a]χ^2 were estimated using full maximum likelihood.

[b] R^2 represents in the variance explained in comparison to model 1.

***p*<.01, ****p*<.001.

Table 17. Model estimates for ECBQ Attention Shifting.

	Model 1		Model 2	
**Fixed Components**	Est.	*SE*	Est.	*SE*
Intercept	4.107	.127	3.742	.357
Age	.023***	.004	.023***	.004
Gender	-.005	.042	-.003	.043
Group-Mean Centered Gentle			.050	.029
Group-Mean Centered Active			.039	.031
Group Mean Gentle			.016	.123
Group Mean Active			.341**	.122
**Variance Components**				
**Within**	.373	.018	.377	.019
**Between**				
Intercept	.026	.013	.015	.009
Slope				
**Model Fit**				
χ^{2a}	1621.47		1575.16	
AIC	1649.63		1627.15	
BIC	1673.44		1669.76	
R^2 within (%)[b]			.00	
R^2 between (%)[b]			42.31	

Notes: Est = Estimate. *SE* = Standard Error

[a]χ^2 were estimated using full maximum likelihood.

[b] R^2 represents in the variance explained in comparison to model 1.

***p*<.01, ****p*<.001.

Table 18. Model estimates for ECBQ Cuddliness.

	Model 1		Model 2	
**Fixed Components**	Est.	*SE*	Est.	*SE*
Intercept	4.928	.161	4.044	.546
Age	.025***	.005	.023***	.005
Gender	-.183***	.052	-.173**	.053
Group-Mean Centered Gentle			.146***	.036
Group-Mean Centered Active			-.023	.038
Group Mean Gentle			.289	.193
Group Mean Active			.203	.191
**Variance Components**				
**Within**	.577	.028	.578	.028
**Between**				
Intercept	.051	.024	.044	.023
Slope				
**Model Fit**				
χ^{2a}	2002.07		1941.90	
AIC	2028.72		1989.48	
BIC	2052.53		2032.09	
R^2 within (%)[b]			.00	
R^2 between (%)[b]			13.72	

Notes: Est = Estimate. *SE* = Standard Error

[a]χ^2 were estimated using full maximum likelihood.

[b] R^2 represents in the variance explained in comparison to model 1.

**p<.01, ***p<.001.

Table 19. Model estimates for ECBQ Inhibition.

	Model 1		Model 2	
**Fixed Components**	Est.	*SE*	Est.	*SE*
Intercept	3.369	.186	3.308	.730
Age	.032***	.005	.030***	.005
Gender	-.218***	.060	-.226***	.061
Group-Mean Centered Gentle			.124**	.042
Group-Mean Centered Active			-.027	.044
Group Mean Gentle			.006	.261
Group Mean Active			.109	.258
**Variance Components**				
**Within**	.760	.037	.769	.038
**Between**				
Intercept	.071	.033	.085	.042
Slope				
**Model Fit**				
χ^{2a}	2241.00		2186.92	
AIC	2266.76		2231.53	
BIC	2290.57		2274.14	
R^2 within (%)[b]			.00	
R^2 between (%)[b]			.00	

Notes: Est = Estimate. *SE* = Standard Error

[a]χ^2 were estimated using full maximum likelihood.

[b] R^2 represents in the variance explained in comparison to model 1.

***p*<.01, ****p*<.001.

Table 20. Model estimates for ECBQ Low-Intensity Pleasure.

	Model 1		Model 2	
**Fixed Components**	Est.	*SE*	Est.	*SE*
Intercept	5.009	.153	3.869	.431
Age	.006	.004	.004	.004
Gender	-.136**	.050	-.121*	.049
Group-Mean Centered Gentle			.234***	.034
Group-Mean Centered Active			.028	.036
Group Mean Gentle			.411**	.149
Group Mean Active			.106	.148
**Variance Components**				
**Within**	.533	.03	.508	.025
**Between**				
Intercept	.040	.019	.024	.014
Slope				
**Model Fit**				
χ^{2a}	1931.45		1827.64	
AIC	1958.47		1877.26	
BIC	1982.28		1919.87	
R^2 within (%)[b]			4.69	
R^2 between (%)[b]			40.00	

Notes: Est = Estimate. *SE* = Standard Error

[a]χ^2 were estimated using full maximum likelihood.

[b] R^2 represents in the variance explained in comparison to model 1.

**p*<.05, ***p*<.01, ****p*<.001.
